# Recent Updates in Neurosurgical Interventions for Spontaneous Intracerebral Hemorrhage: Minimally Invasive Surgery to Improve Surgical Performance

**DOI:** 10.3389/fneur.2021.703189

**Published:** 2021-07-19

**Authors:** Hitoshi Kobata, Naokado Ikeda

**Affiliations:** ^1^Department of Neurosurgery, Osaka Mishima Emergency Critical Care Center, Takatsuki, Japan; ^2^Department of Neurosurgery, Osaka Medical and Pharmaceutical University, Takatsuki, Japan

**Keywords:** intracerebral hemorrhage, minimally invasive surgery, endoscopic surgery, stereotactic surgery, thrombolysis, surgical performance

## Abstract

The efficacy and safety of surgical treatment for intracerebral hemorrhage (ICH) have long been subjects of investigation and debate. The recent results of the minimally invasive surgery plus alteplase for intracerebral hemorrhage evacuation (MISTIE) III trial demonstrated the safety of the procedure and a reduction in mortality compared to medical treatment. Although no improvement in functional outcomes was shown, the trial elucidated that benefits of intervention depend on surgical performance: a greater ICH reduction, defined as ≤ 15 mL end of treatment ICH volume or ≥70% volume reduction, correlated with significant functional improvement. Recent meta-analyses suggested the benefits of neurosurgical hematoma evacuation, especially when performed earlier and done using minimally invasive procedures. In MISTIE III, to confirm hemostasis and reduce the risk of rebleeding, the mean time from onset to surgery and treatment completion took 47 and 123 h, respectively. Theoretically, the earlier the hematoma is removed, the better the outcome. Therefore, a higher rate of hematoma reduction within an earlier time course may be beneficial. Neuroendoscopic surgery enables less invasive removal of ICH under direct visualization. Minimally invasive procedures have continued to evolve with the support of advanced guidance systems and devices in favor of better surgical performance. Ongoing randomized controlled trials utilizing emerging minimally invasive techniques, such as the Early Minimally Invasive Removal of Intra Cerebral Hemorrhage (ENRICH) trial, Minimally Invasive Endoscopic Surgical Treatment with Apollo/Artemis in Patients with Brain Hemorrhage (INVEST) trial, and the Dutch Intracerebral Hemorrhage Surgery Trial (DIST), may provide significant information on the optimal treatment for ICH.

## Introduction

Spontaneous intracerebral hemorrhage (ICH) is the second most common but most devastating type of stroke (1). Although medical and surgical interventions have been developed for the condition, ICH remains a significant cause of death and mortality worldwide (2).

ICH leads to time-dependent progression of brain injury. The initial bleeding causes physical disruption of the cellular architecture of the brain. The hematoma mass can induce intracranial pressure elevation, leading to ischemia and brain herniation. Secondary injury after ICH could be caused by a cascade of events initiated by the primary injury; by the physiological response to the hematoma, such as inflammation; and by the release of clot components such as hemoglobin and iron (3). Theoretically, timely surgical intervention can be effective if a surgery-related brain injury is less severe than that caused by hematoma *per se*.

Consequently, surgery to reduce hematoma volume has been repeatedly evaluated in single-center studies and multicenter randomized controlled trials (RCTs). The International Surgical Trial in Intracerebral Hemorrhage (STICH) was designed to compare early surgery with initial conservative treatment for supratentorial ICH. In this study, 1,033 patients from 83 centers in 27 countries were randomized to receive either early surgery or initial conservative treatment. No significant difference was found in good outcomes between the surgical arm (26%) and the medical arm (24%). However, subgroup analysis suggested that surgery might benefit in patients with lobar hemorrhages within 1 cm of the cortical surface (4). Based on these findings, the STICH II trial was undertaken to assess the effectiveness of early surgery vs. medical management for patients with superficial lobar ICH of 10–100 mL without intraventricular hemorrhage. The rates of favorable outcomes were 41% and 38% in the surgical and medical arms, respectively, with no significant difference (5). However, the results of the STICH trials may not be generalizable because of the high rate of patients' crossover from the medical arm to the surgical arm. Additionally, comatose patients and patients at risk of cerebral herniation were excluded.

To date, surgery did not demonstrate a clear benefit compared to conservative treatment. According to the current guidelines, supratentorial hematoma evacuation might be considered as a life-saving measure, and decompression with or without evacuation might reduce mortality in comatose supratentorial ICH patients with large hematomas. Outcomes with performing minimally invasive clot evacuation using stereotactic or endoscopic aspiration with or without thrombolytic usage were deemed uncertain (6).

Minimally invasive surgery (MIS) with thrombolysis in ICH evacuation (MISTIE) was designed to minimize craniotomy-related secondary brain injury associated with conventional surgical procedures. The procedure was rigorously standardized and electrocautery and mechanical manipulations of brain tissue were eliminated. The recent results of MISTIE III trial have raised a great deal of interest regarding the indications for ICH surgery. Although MISTIE III did not demonstrate a positive effect on functional outcomes compared with standard medical care for ICH patients, it presented an essential insight into surgery for ICH (7).

## Lessons Learned From the MISTIE Trial

The MISTIE procedure involves stereotactic hematoma evacuation followed by residual clot lysis with alteplase. After manual aspiration of the clot with a syringe, the surgeons insert a soft drainage catheter to facilitate up to nine injections of alteplase and passive clot drainage. The goals of the intervention were to decrease the clot size to <15 mL at the end of treatment (EOT) or stop when a maximum of nine doses of alteplase (1 mg every 8 h) was administered. This procedure seemed safe in the phase 2 study, with a possible advantage of having better functional outcomes compared to medical treatment (8). MISTIE III was a randomized, controlled, open-label, blinded endpoint, phase 3 explanatory trial of image-guided, catheter-based removal of an ICH of 30 mL or more. Seventy-eight hospitals in the USA, Canada, Europe, Australia, and Asia participated in the trial, including 499 patients (250 in the MISTIE group and 249 in the standard care group).

The mean reduction in hematoma size was 69% (SD 20), and the mean end-of-treatment (EOT) volume was 16 mL (SD 13) in the MISTIE group. The trial failed to reach the primary endpoint of improved functional outcomes; 110 (44%) of 249 patients in the MISTIE group and 100 (42%) of 240 patients in the standard medical care group had a modified Rankin Scale (mRS) score of 0–3 at 365 days (adjusted risk difference, 4%; 95% CI: −4–12; *P* = 0.33). However, the secondary endpoints indicate acceptable safety and a slight decrease in mortality in the MISTIE group, with a hazard ratio of 0.67 (95% CI: 0.45–0.98; *p* = 0.037) (7).

Most importantly, MISTIE III was the first to examine surgical performance in association with outcomes. The as-treated analysis demonstrated that a more significant ICH reduction has a higher likelihood of achieving an mRS of 0–3 with a minimum evacuation threshold of ≤ 15 mL EOT ICH volume or ≥70% volume reduction when controlling for disease severity factors. Mortality benefit was achieved at ≤ 30 mL EOT ICH volume or >53% volume reduction. Moreover, each additional milliliter removed beyond 70% led to a 6% improvement in the chance of achieving a good outcome of mRS 0–3 (OR = 1.06, 95% CI: 1.02–1.10, *P* = 0.002). In addition, surgeon and site case experiences were related to ICH evacuation efficacy. There was a threshold of four prior MISTIE trial cases by the surgeon and seven prior cases by the site, above which there were no cases with poor (>30 mL) EOT ICH (9). In an aim to improve surgical performance and maximize its benefits, a guide to the surgical protocols for MISTIE and CLEAR (Clot Lysis: Evaluating Accelerated Resolution of Intraventricular Hemorrhage) (10) has been recently published, and this also includes tutorial videos (11).

These findings led to a re-evaluation of the STICH trials (4, 5). In the analysis of lobar hemorrhages in MISTIE III and STICH II, EOT ICH volume ≤ 28.8 mL in MISTIE III and ≤ 30.0 mL in STICH II showed increased probability of an mRS of 0–3 at 180 days (*P* = 0.01 and 0.003, respectively). Two different interventions for ICH evacuation showed similar threshold EOT volumes for good outcomes (12).

In MISTIE III, several issues must be considered. Patients were randomized when active bleeding was stopped. Consequently, the mean time from onset to enrollment, to surgery, to the first dose of alteplase, and to the end of treatment was delayed for 47.0, 58.3, 72.6, and 123 h, respectively. This time course seems suboptimal for the management of ICH, because studies in animal models suggest that the time window for hematoma evacuation is 6–12 h (13). Irregularly shaped hematomas were significantly associated with less efficient ICH removal. The thalamic ICH (“Trajectory B” per trial protocol) was most likely associated with catheter malposition and poor evacuation efficacy (9, 14). Clot lysis may be insufficient in such cases. Life-threatening ICHs were excluded (7). Urokinase-type plasminogen activator may be more beneficial than alteplase (15). Fibrinolysis may be enhanced by Doppler sonography (16).

## Endoscopic Surgery

Neuroendoscopic surgery enables the removal of ICH under direct visualization in a less invasive manner than conventional craniotomy. In contrast to the stereotactic procedure, hemostasis at the bleeding point is also possible. ICES (Intraoperative Stereotactic Computed Tomography-Guided Endoscopic Surgery), a multicenter RCT, showed considerable safety and efficacy (17). Advantages of neuroendoscopic surgery over conventional craniotomy include a higher hematoma evacuation rate, shorter operation time, less intraoperative blood loss, better neurological outcomes, and shorter hospital stay (18). Its hematoma evacuation rate has been reported to reach around 90% (18, 19). Tips for safe and effective endoscopic clot evacuation are presented in schematic drawings (19). Endoscopic hematoma evacuation without decompression was safe and effective, even in patients with large putaminal ICH (20). The effectiveness of endoscopic surgery for large, life-threatening ICH has also been reported (21). It is unlikely that a large decompressive craniectomy would be required when the endoscopic procedure was successfully and timely achieved. With the development of endoscope technology and the accumulation of therapeutic experience, endoscopic evacuation will become more widely employed.

**Figure 1:**
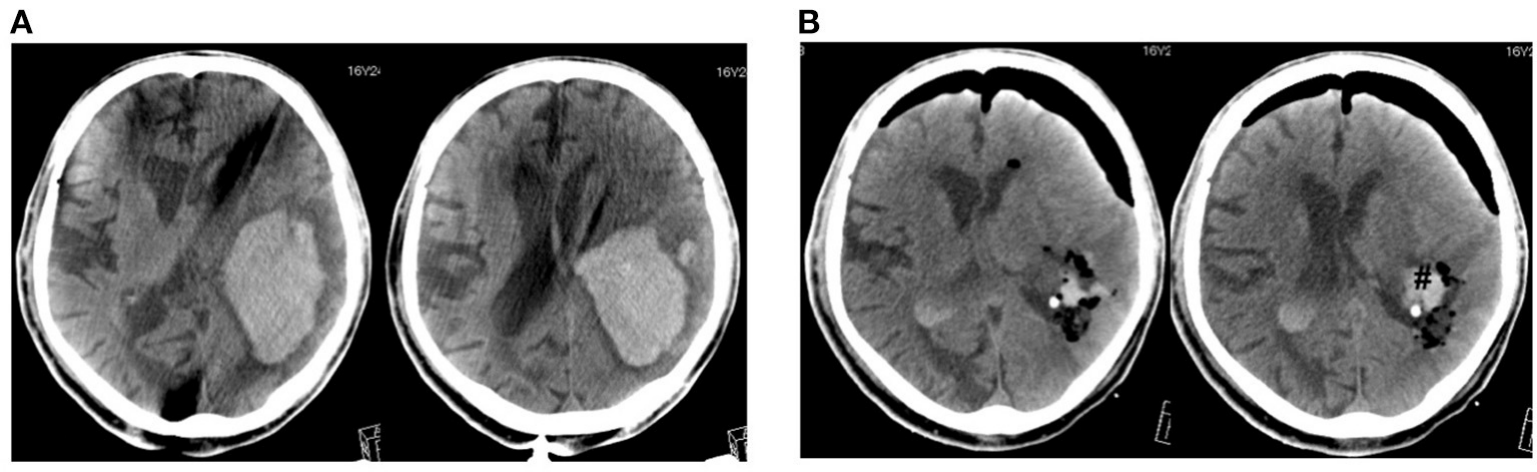
A case of large lobar hemorrhage (110 ml). CT scans before **(A)** and after operation **(B)**. ^#^Hematoma remnant.

**Figure 2:**
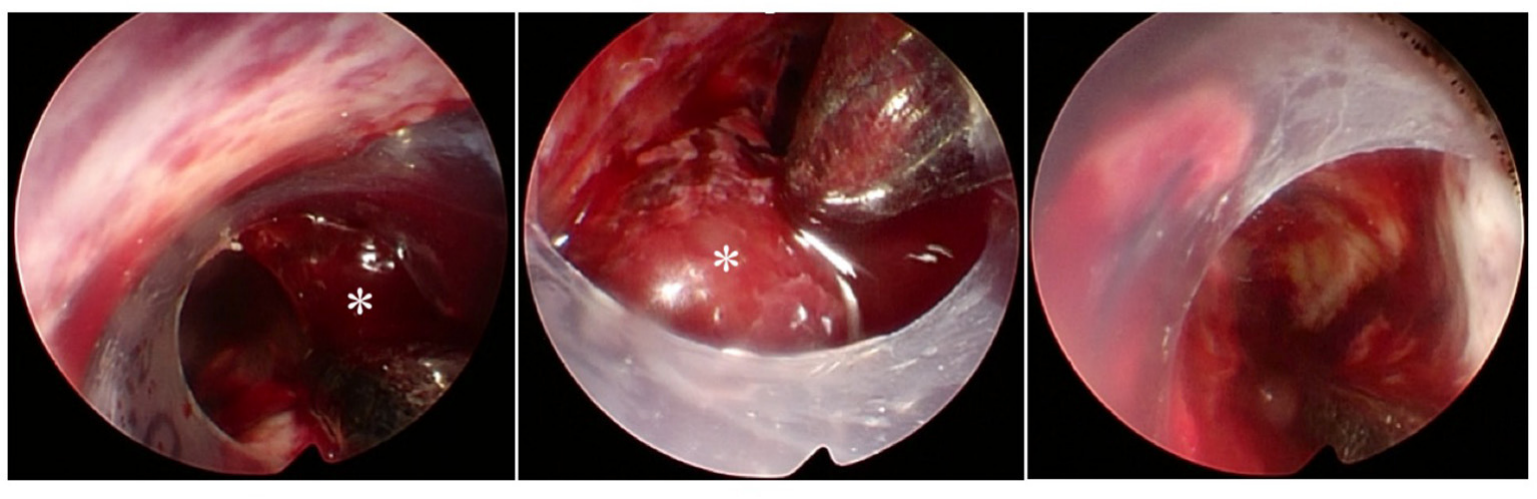
Endoscopic view through a transparent sheath inserting to the hematoma cavity. *Hard clot difficult to suction.

Although endoscopic ICH evacuation seems promising, there are some concerns. In conventional craniotomy, the CT angiography spot sign is associated with increased intraoperative bleeding, more postoperative rebleeding, and larger residual ICH volumes (22). Likewise, in endoscopic surgery, multivariate analysis revealed that the spot sign was the only independent predictor of postoperative recurrent hemorrhage and a significant risk factor for intraoperative bleeding (23). Thus, extra effort and treatment are needed to manage ICH patients with the spot sign (23). The bleeding point should be identified under the guidance of the navigation system and coagulated for hemostasis. To avoid intraoperative and postoperative bleeding, intentional preservation of hard clots may be safer (Figures 1, 2). It is important to recognize the patient factors against endoscopic surgery. Poor evacuation rate was seen in patients with chronic renal failure who were treated with hemodialysis, as well as patients with liver cirrhosis (24). Hematologic diseases and the use of antithrombotics also adversely affect the surgical outcomes (22).

## Recent Meta-Analyses

The optimal treatment of ICH is of great concern in the field of neurosurgery/neurointensive care. Earlier studies comparing conventional craniotomy with the best medical management failed to show a clear benefit (4, 5). More recent experiences with MIS have shown greater promise. Table 1 summarizes recent meta-analyses on MIS for ICH (25–33). Two latest studies (26, 27) include the results of MISTIE III. Surgical treatment of ICH, particularly with MIS, is beneficial compared to medical treatment. Compared with craniotomy, MIS is associated with fewer deaths (28, 31, 32), rebleeding (28, 31) and complications (29–31), and more favorable outcomes (28, 31, 32). The latest meta-analysis and trial sequential analysis confirmed the benefits of MIS over conservative treatment (26). Moreover, neuroendoscopic surgery is superior to craniotomy or other treatments in terms of good functional outcomes (25, 32, 33), mortality (25, 32, 33), hematoma evacuation rate (25), blood loss (25), complications (29, 30, 33), operation time (25), hospital stay (25), and ICU stay (25).

**Table 1:** Summary of recent meta-analyses on minimally invasive surgery for intracerebral hemorrhage.

**Study (search period)**	**Patients number**	**Trials included**	**Treatment compared**	**Outcomes**	**Risk/Odds Ratio (95% CI)**	
Sun et al. (25) (up to June 2019)	1,506	8	NE vs. CT	Good functional outcome	OR	3.27 (1.73, 6.21)
	1,859	15		Mortality		0.43 (0.32, 0.58)
	883	10		Hematoma evacuation rate		8.14 (3.46, 12.83)
	781	7		Blood loss volume		−294.77 (−494.61, −94.93)
	1,060	13		Operation time		−99.03 (−119.56, −78.50)
	287	3		Hospital stay		−2.32 (−3.96, −0.68)
	157	3		ICU stay		−4.35 (−6.45, −2.26)
Zhou et al. (26) (up to March 2019)	1,955	11	MIS vs. conservative	Significant neurological debilitation or death	RR	0.82 (0.72, 0.94)
	1,955	11		Death		0.74 (0.62, 0.88)
Sontag et al. (27) (up to February 21, 2019)	3,886	20	Any surgery vs. medical	Good functional outcome	RR	1.40 (1.22, 1.60)
	2,045	20	MIS vs. medical			1.47 (1.26, 1.72)
	2,133	4	Any surgery vs. medical^*^			1.10 (0.98, 1.25)
Xia, et al. (28) (up to April 2018)	2,466	14	MIS vs. CT	Mortality	RR	0.76 (0.60, 0.97)
	1,273	10		Rebleeding		0.42 (0.19, 0.95)
	1,858	6		Good recovery		2.27 (1.34, 3.83)
Nam and Kim (29) (up to December 2017)	295	3	NE vs. CT	Death	OR	0.56 (0.24, 1.31)
	295	3		Complication		0.11 (0.03, 0.20)
Zhao et al. (30) (up to December 2017)	295	3	NE vs. CT	Death	RR	0.58 (0.26, 1.29)
	295	3		Complication		0.11 (0.06, 0.20)
Tang et al. (31) (up to November 2017)	258	4	MIS vs. conservative	GOS score	RR	1.55 (1.21, 1.97)
	352	5	MIS vs. CT			1.69 (1.10, 2.59)
	282	4	MIS vs. conservative	Pulmonary infection		1.26 (1.13, 1.40)
	486	3	MIS vs. CT			0.47 (0.26, 0.83)
	600	6	MIS vs. conservative	Mortality		0.26 (0.17, 0.40)
	1,127	8	MIS vs. CT			0.84 (0.65, 1.09)
	696	4	MIS vs. CT	ADL score		1.26 (1.13, 1.40)
	745	6	MIS vs. CT	Rebleeding		0.47 (0.26, 0.83)
Scaggiante et al. (32) (up to October 2017)	2,152	15	MIS vs. other treatment	Significant neurological debilitation or death	OR	0.46 (0.36, 0.57)
	863	5	MIS vs. CT			0.44 (0.29, 0.67)
	384	5	NE vs. other treatment			0.40 (0.25, 0.66)
	1,526	8	SE vs. other treatment			0.47 (0.34, 0.65)
	2,086	14	MIS vs. other treatment	Death		0.59 (0.45, 0.76)
	797	5	MIS vs.CT			0.56 (0.37, 0.84)
	384	5	NE vs. other treatment			0.37 (0.20, 0.67)
	1,404	7	SE vs. other treatment			0.76 (0.56, 1.04)
Yao et al. (33) (up to October 2017)	1,213	18	NE vs. other treatment	Mortality	RR	0.61 (0.48, 0.78)
	721	10		GOS 1–3, mRS 4–6		0.78 (0.70, 0.87)
	881	13		Rebleeding		0.40 (0.23, 0.69)
	641	8		Pneumonia		0.42 (0.28, 0.61)
	364	4		Meningitis		0.52 (0.16, 1.70)
	395	3		Epilepsy		0.58 (0.32, 1.05)
	451	4		Digestive disease		1.27 (0.75, 2.15)

* *High quality studies only*.

*NS, neuroendoscopy; MIS, minimally invasive surgery; SE, stereotactic evacuation; CT, craniotomy; GOS, Glasgow Outcome Scale; mRS, modified Rankin Scale; OR, Odds ratio; RR, Risk ratio*.

## Emerging Minimally Invasive Techniques for ICH Evacuation and Ongoing Studies

MIS procedures have continued to evolve with the support of advanced guidance systems and devices in favor of the better surgical performance of ICH removal. In summary, the hematoma is removed by newly developed aspirators through a narrow tubular retractor to mitigate brain injury. New devices can immediately evacuate the hematoma at the time of the procedure without the need for prolonged thrombolytic irrigation (34–36). Several RCTs are underway using newly developed instruments and techniques for MIS (Table 2). These studies include protocols aimed at active clot removal at an earlier phase (<8 h).

**Table 2:** Ongoing studies of minimally invasive surgery for intracerebral hemorrhage.

Study^*^	**Study type**	**Intervention**	**Primary endpoint**	**Patients number**	**Time window**	**Study start point**	**Estimated study completion point**
ENRICH	Randomized	NICO BrainPath and Myriad	Functional improvement (mRS)	300	<24 h	December 2016	December 2021
INVEST	Single arm	Apollo System	Rate of recruitment/successful follow up obtainment	50	<72h	June 30, 2017	June 2021
MIND	Randomized	Artemis Neuro Evacuation Devices	Global disability (mRS)/Mortality	500	<72 h	February 7, 2018	July 2025
DIST	Non-randomized	Artemis Neuro Evacuation Devices	Death/Neurological deterioration/Proportion of volume reduction	400	<8 h	December 3, 2018	February 2021
EVACUATE	Randomized	Aurora Surgiscope System	mRS	240	<8 h	September, 2020	December, 2026
MIRROR	Observational	Aurora Surgiscope System	Rate of Surgical Success (reduction to <15 ml)	500	<12 h	October, 2020	October, 2029

* *Official title of the study*.

*ENRICH, A Multi-center, Randomized, Clinical Trial Comparing Standard Medical Management to Early Surgical Hematoma Evacuation Using Minimally Invasive Parafascicular Surgery (MIPS) in the Treatment of Intracerebral Hemorrhage (ICH)*.

*INVEST, A Single Arm, Feasibility Study of Minimally Invasive Endoscopic Surgical Treatment with Apollo for Supratentorial Intracerebral Hemorrhage (ICH)*.

*MIND, A Prospective, Multicenter Study of Artemis: A Minimally Invasive Neuro Evacuation Device in the Removal of Intracerebral Hemorrhage*.

*DIST, The Dutch Intracerebral Hemorrhage Surgery Trial Pilot Study: Minimally-invasive Endoscopy-guided Surgery for Spontaneous Intracerebral Hemorrhage*.

*EVACUATE, Ultra-Early, Minimally inVAsive intraCerebral Hemorrhage evacUATion vs. Standard treatment*.

*MIRROR, Minimally Invasive IntRaceRebral HemORrhage Evacuation*.

*mRS, modified Rankin Scale*.

**Figure 3:**
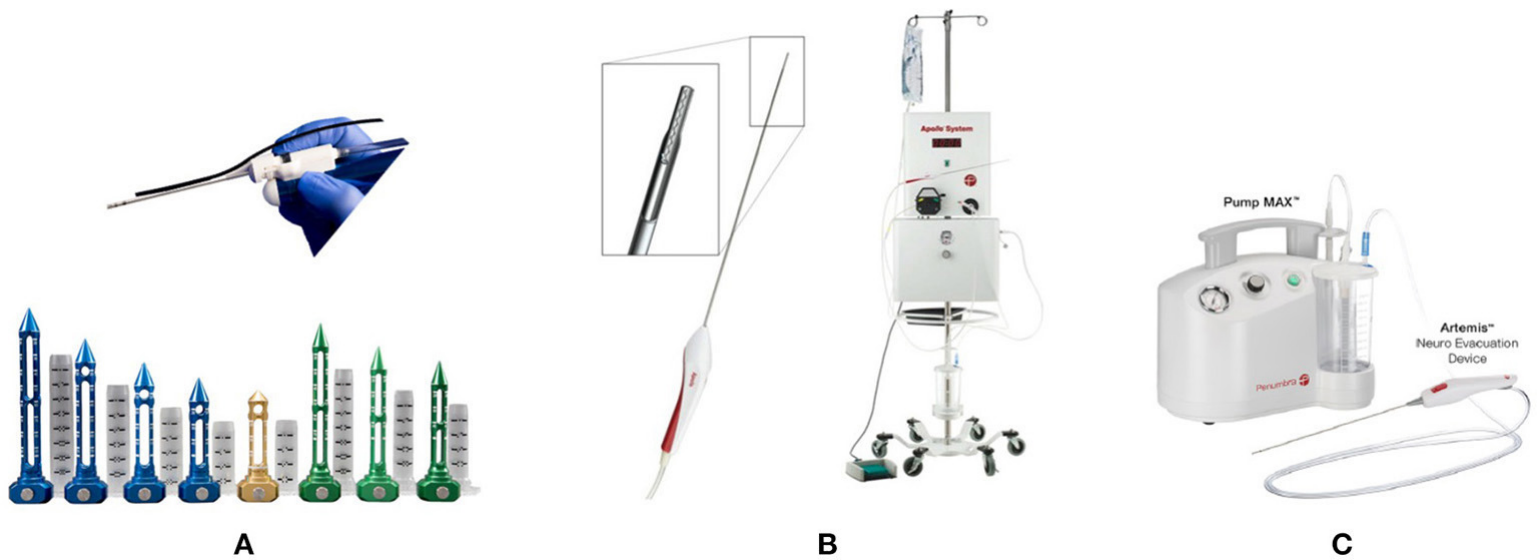
Emerging minimally invasive instruments. **(A)** NICO BrainPath system and myriad handpiece (NICO Corp, Indianapolis, IN, USA). **(B)** The Apollo system. The Wand and aspiration–irrigation system (Penumbra Inc, Alameda, CA, USA). **(C)** The Artemis Neuro Evacuation Device and Pump MAX™ aspiration system (Penumbra, Alameda, CA, USA).

Endoport-mediated evacuation is an active evacuation technique that utilizes the BrainPath endoport (NICO Corp., Indianapolis, IN, USA). BrainPath consists of an access sheath of 11.0 or 13.5 mm in diameter and multiple lengths (50, 60, 75, and 95 mm) and an internal obturator (Figure 3A). The BrainPath is placed through a small craniotomy (2–3 cm). The opening may be planned with magnetic resonance tractography to facilitate the least traumatic trans-sulcal access to the lesion. Once the sheath is placed stereotactically, the obturator is removed, the clot is evacuated using standard microsurgical techniques. Active bleeding could be identified and controlled. A Myriad handpiece, which is an automated and nonablative resection device (NICO Corp, Indianapolis, IN, USA), can be used when necessary, as in cases of high-density clots. In 39 consecutive patients treated with the NICO device, a clot reduction rate of ≥90% was achieved in 72% of the patients (37). The Early MiNimally-invasive Removal of IntraCerebral Hemorrhage (**ENRICH**) trial, which makes use of Brain Path and Myriad, is currently ongoing (https://clinicaltrials.gov/ct2/show/NCT02880878).

The Apollo system (Penumbra Inc., Alameda, CA, USA) is composed of an aspiration–irrigation system that allows the removal of hematoma through a low-profile wand for the controlled aspiration of soft tissue and fluid (Figure 3B). A vibrational element housed within the wand vibrates at a high frequency to break down the clot inside the wand and prevent clogging. No energy is transferred to the tissue outside the device (38). Early experiences with the Apollo system indicate the effectiveness of this device (39). The Minimally Invasive Endoscopic Surgical Treatment with Apollo vs. Medical Management for Supratentorial ICH (**INVEST**) trial is currently underway as a phase II study (https://clinicaltrials.gov/ct2/show/NCT02654015).

The Artemis System (Penumbra, Alameda, CA, USA) is intended for the controlled aspiration of tissue and/or fluid from the ventricular system and/or cerebrum. The device works in conjunction with a neuroendoscope through a 19 F (6 mm) sheath. Together with the Pump MAX™ aspiration system, Artemis offers powerful and controlled evacuation (Figure 3C). Stereotactic ICH Underwater Blood Aspiration (**SCUBA**) is an endoscopic evacuation technique that uses Apollo/Artemis devices (40). This procedure could be safely indicated for patients with ICH who showed spot signs or hematoma. Two studies utilizing the Artemis System, namely the Dutch Intracerebral Hemorrhage Surgery Trial (**DIST**) (https://clinicaltrials.gov/ct2/show/NCT03608423) and Artemis in the removal of intracerebral hemorrhage (**MIND**), are currently ongoing (https://clinicaltrials.gov/ct2/show/NCT03342664).

The Aurora Surgiscope System designed by Rebound Therapeutics is the first disposable, single-use endoscope with an outer diameter of 11.5 mm. The first clinical trials of this device, namely the Ultra-Early, Minimally inVAsive intracerebral hemorrhage evacUATion vs. Standard Treatment (**EVACUATE**) (https://www.clinicaltrials.gov/ct2/show/NCT04434807) and Minimally Invasive IntRaceRebral HemORrhage Evacuation (**MIRROR**) (https://clinicaltrials.gov/ct2/show/NCT04494295), began enrolling patients in 2020.

## Significance of Conventional Craniotomy

**Figure 4:**
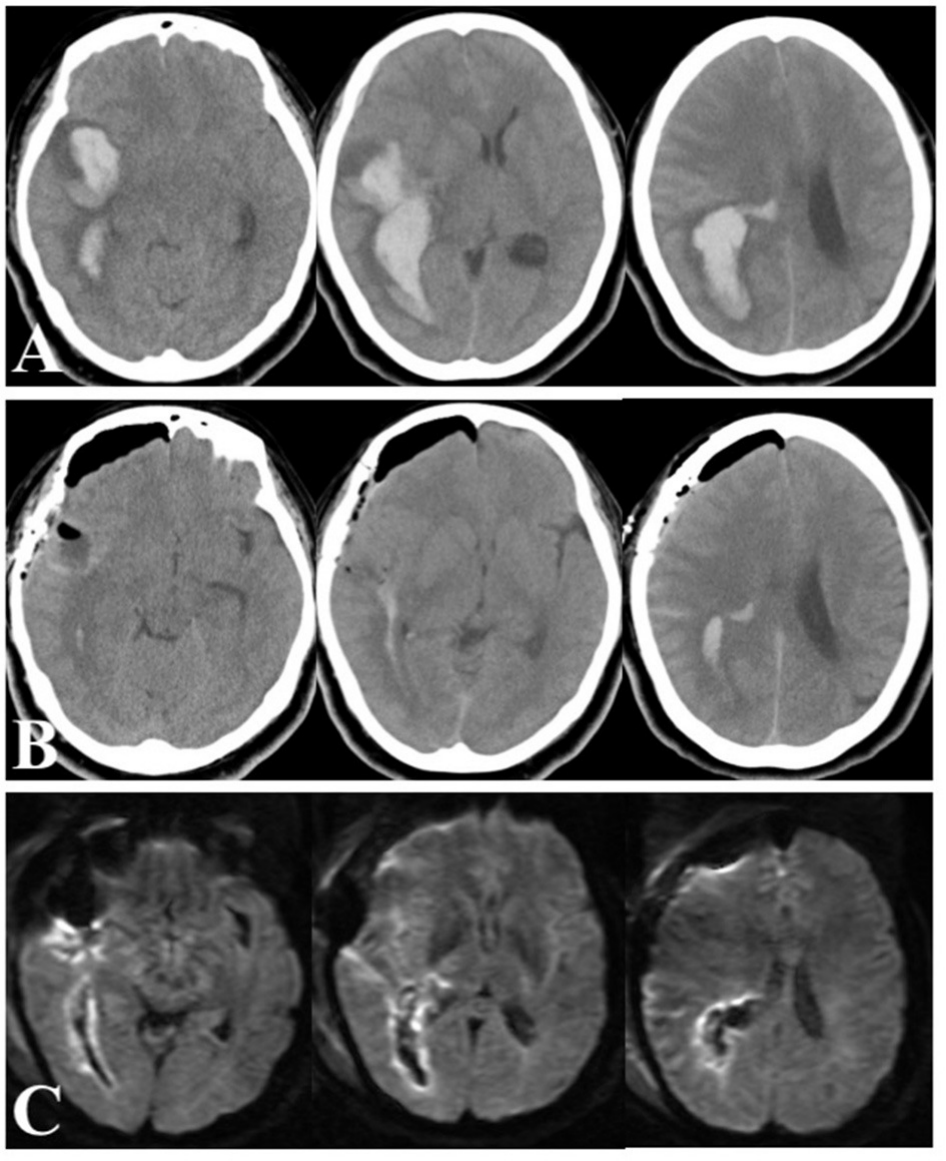
Head CT scans on arrival **(A)**, immediately after ICH removal **(B)**, and diffusion-weighted MRI images next day **(C)**.

The role of early ICH evacuation remains a topic of debate. Craniotomy for ICH evacuation remains a life-saving measure in critical situations, although it is difficult to standardize. Craniotomy allows secure hemostasis, multidirectional trajectories for evacuation, and external decompression to control intracranial pressure when necessary. Herein, we also present a case of a 49-year-old woman who arrived at the emergency room 1 h after ictus. Her Glasgow Coma Scale score was 7 (E1V1M5), and head CT scans showed an irregular, lobulated ICH of 66 mL in the right temporal lobe (Figure 4A). She was transferred to the operating room 1 h after arrival. A craniotomy of 4.5 cm in diameter was made, and a 1.5 cm corticotomy was performed. Hematoma evacuation was completed 3.5 h after ictus. Postoperative CT images showed >90% removal of the hematoma (Figure 4B), and diffusion-weighted MR images taken the next day demonstrated limited high-intensity lesions around the hematoma (Figure 4C). She had a remarkable recovery, showing no apparent paresis or disturbed consciousness, but presented with left homonymous hemianopia. Two weeks later, her mRS score was 2, and she was transferred to a rehabilitation hospital. In this case, MIS may carry a higher risk of rebleeding and appeared to be disadvantageous because of the shape and location of the ICH. Open craniotomy allowed meticulous microsurgical manipulation *via* multidirectional trajectories with freely changeable directions for this complex-shaped hematoma. Of note, it is essential to minimize the use of electrocautery and brain compression during the procedure.

A meta-analysis indicated improved outcomes with surgery if undertaken within 8 h of ictus (41), whereas ultra-early craniotomy within 4 h from ictus was associated with an increased risk of rebleeding (42). The optimal timing for surgery depends on the balance between the initial hematoma size, the risk of rebleeding, and secondary injury such as peripheral edema caused by the hematoma. In animal experiments, the pathophysiological time window of minimally invasive procedures for hematoma evacuation might be 6–12 h after hemorrhage (13). A systemic review reported the optimal time window for ICH evacuation to be 7–24 h after ictus (43). Since individual patients have different pathological conditions, it may be difficult to generalize the optimal timing of surgical procedures. Craniotomy should further be maintained as an option.

The potential benefits of craniotomy have been reported (44). Decompressive hemicraniectomy associated with ultrasound-guided minimally invasive puncture and drainage showed a significantly higher survival rate and better functional outcome for deteriorating ICH in the basal ganglia (45). Thus, an individual-based tailored surgical approach may be beneficial.

## Future Perspectives

Advances in therapeutic devices and techniques, especially endovascular thrombectomy, have made a significant contribution to the treatment of acute cerebral ischemia. Likewise, recent advances in therapeutic devices are making great strides in the treatment of ICH. Early and optimal treatment for ICH is warranted, by experienced neurosurgeons/neurointensivists, in high-volume centers. It is time to discard the therapeutic nihilism of past days. Although the way forward is still far away, we have every reason to be optimistic for the future of ICH treatment.

## Author Contributions

HK and NI contributed conception of the article. HK wrote the first draft of the article. All authors approved the submitted version.

## Conflict of Interest

The authors declare that the research was conducted in the absence of any commercial or financial relationships that could be construed as a potential conflict of interest.
